# Knowledge, attitudes, and practices regarding occupational protection among nursing assistants: a cross-sectional survey with K-means clustering analysis

**DOI:** 10.3389/fpubh.2026.1807345

**Published:** 2026-04-28

**Authors:** Lishan Huang, Jialin Zhang, Wenting Huang, Lidan Ye, Xiaoquan Li, Jiayi Han, Lin Wang, Jianyun Li

**Affiliations:** The Affiliated Guangdong Second Provincial General Hospital of Jinan University, Guangzhou, China

**Keywords:** associated factors, K-means clustering, knowledge, attitudes, and practices (KAP), nursing assistants, occupational protection

## Abstract

**Background:**

Nursing assistants are routinely exposed to occupational hazards; this exposure, coupled with insufficient awareness and inconsistent compliance with safety precautions, significantly increases their risk of infection. This study aimed to assess the knowledge, attitudes, and practices (KAP) regarding occupational protection among nursing assistants in China.

**Methods:**

We conducted a cross-sectional study from October to November 2025. We recruited 505 nursing assistants from 20 general hospitals in Guangdong Province using a convenience sampling approach. Data were collected via a self-administered questionnaire consisting of two parts: a socio-demographic section and the Occupational Protection KAP Assessment Scale. To identify distinct KAP profiles and their associated factors, we first performed cluster analysis, followed by univariate analysis and multinomial logistic regression.

**Results:**

We analyzed 503 valid questionnaires, yielding an effective response rate of 99.6%. The mean scores (±SD) for knowledge, attitudes, and practices dimensions were 13.78 ± 1.71, 65.90 ± 5.54, and 63.23 ± 8.80, respectively, with score rates of 91.9, 87.9, and 84.3%. Cluster analysis revealed four distinct KAP profiles: the Synergistic Group (47.3%), Weak KAP Group (10.1%), High-Knowledge–Low-Attitude–Weak-Practice Group (18.7%), and High-Knowledge–High-Attitude–Low-Practice Group (23.9%). Significant differences (*p* < 0.05) were observed among the clusters in terms of healthcare institution type and possession of a professional qualification certificate.

**Conclusion:**

Nursing assistants demonstrated good knowledge of occupational protection, but there was a notable gap in practical implementation. The four KAP types identified through cluster analysis showed high heterogeneity. Targeted interventions should be developed based on these distinct profiles, shifting the focus from individual training to the establishment of systematic and supportive organizational environments and management systems. This approach can provide an empirical basis for optimizing occupational health policies for nursing assistants in China.

## 1 Introduction

China’s rapidly aging population (1), compounded by evolving family structures and growing older population care needs (2), is driving a sustained increase in the demand for nursing assistants. Nursing assistants provide daily living care for patients and perform auxiliary tasks under the guidance of medical professionals. They thus constitute a vital component of the medical auxiliary workforce, extending clinical services and supplementing the nursing assistants (3). However, their frontline role exposes them to a spectrum of occupational hazards, including physical, chemical, and biological risks (4, 5), resulting in significant occupational safety concerns. Due to direct contact with bodily fluids, frequent handling of sharp instruments, and patient handling tasks, this group exhibits a high occupational injury rate of 43.41%, as evidenced by research from Chaves et al. (6). Recent national policies—including the Notice on Strengthening the Training and Standardized Management of Healthcare Assistants (7) and the Notice on Issuing the Pilot Work Plan for Caregiver-Free Hospital Services (8)—have explicitly mandated the standardization of occupational protection for this workforce. With the pilot implementation of the “unaccompanied care model,” the workload and exposure risks for nursing assistants are projected to rise, rendering research on their occupational protection imperative. Furthermore, the occupational safety challenges faced by nursing assistants, particularly those related to precarious employment, training gaps, and the translation of knowledge into practice, are issues of global concern in healthcare systems worldwide (9).

However, current research on occupational protection primarily focuses on physicians and nurses (10–12), leaving a significant gap in studies specifically targeting healthcare assistants. The Knowledge-Attitude-Practice (KAP) framework posits that health-related behaviors are shaped through a sequence of stages: knowledge acquisition, attitude formation, and behavioral adoption (13). This framework provides a foundational model for studying occupational protection behaviors. This group, however, exhibits distinct occupational characteristics, including frequent employment through third-party agencies, high job mobility (14), fragmented training (15), and resource access contingent on institutional allocation (14). These factors may create structural barriers that disrupt the linear “knowledge-attitude-practice” pathway assumed by the standard KAP model for this workforce.

Compared to conventional methods that rely on aggregated KAP scores or linear regression modeling, the primary strength of K-means clustering lies in its ability to uncover intrinsic heterogeneity within the target population, rather than treating it as a homogeneous cohort (16). This approach enables the identification of latent subgroups characterized by distinct, and often imbalanced, profiles of knowledge, attitude, and practice. Consequently, it provides a robust empirical foundation for developing targeted interventions tailored to the specific characteristics of these subgroups. Therefore, this study aimed to investigate the status of occupational protection KAP among healthcare assistants in 20 general hospitals in Guangdong Province, China. It employed cluster analysis to identify distinct subgroups within this population and to explore the factors influencing their occupational protection levels. The aim was to provide an evidence-based reference for developing targeted interventions and training programs for this critical workforce.

## 2 Materials and methods

### 2.1 Study design and population

This cross-sectional study employed a convenience sampling method to recruit 503 nursing assistants from 20 healthcare institutions in Guangdong Province, China, between October and November 2025. The recruitment was facilitated through third-party agencies. Specifically, the leaders of these agencies distributed online survey links to eligible nursing assistants. A total of 550 invitations were sent out, and 503 valid responses were collected, resulting in a response rate of 91.5%. Eligibility criteria were: (i) age 18 years or older, and (ii) current employment by a hospital or a third-party agency. Exclusion criteria were: (i) absence from duty (e.g., due to leave or illness) during the survey period, or (ii) plans to resign imminently.

The sample size was calculated using the standard formula for cross-sectional studies (17): *n* = (Z^2 × P(1−P))/δ^2. Assuming a population proportion (P) of 0.5, a type I error (*α*) of 0.05, a margin of error (δ) of 0.05, and an anticipated valid response rate of 90%, the minimum required sample size was determined to be 430.

### 2.2 Ethical considerations

The study protocol received approval from the Ethics Committee of Guangdong Second Provincial General Hospital (approval no.: 2025-KY-KZ-363-01). Prior to data collection, all participants provided written informed consent.

### 2.3 Questionnaire introduction

We developed a self-administered questionnaire to assess occupational protection KAP through a literature review and expert consultations. The initial draft was refined based on feedback from three nursing experts to finalize the Chinese version. The final questionnaire consisted of four sections: (1) socio-demographic characteristics and (2–4) the three KAP assessment dimensions.

The collected socio-demographic data included: age (years); gender (male, female); years of experience (<3, 3–5, >5 years); education level (primary school or below, junior high school, high school/vocational school, college or above); institution type (tertiary hospital, secondary hospital); department (internal medicine, surgery, other); average monthly income (<6,000, 6,000–8,000, >8,000 CNY); receipt of systematic occupational protection training (yes, no); and possession of a professional qualification certificate (yes, no).

The KAP assessment section contained 45 items distributed across the three core dimensions: knowledge, attitude, and practice. Items were scored using distinct formats tailored to each dimension. Knowledge items were scored using a binary format (1 for ‘yes’, 0 for ‘no’). Both attitude and practice items used a 5-point Likert scale. For attitude items, anchors ranged from ‘not important at all’ (1 point) to ‘very important’ (5 points). For practice items, anchors ranged from ‘never’ (1 point) to ‘always’ (5 points). The theoretical total score ranged from 30 to 165. The subscale scores ranged as follows: knowledge (0–15), attitude (15–75), and practice (15–75). Higher scores on each scale indicated a more favorable outcome (e.g., greater knowledge, a more positive attitude, or better self-reported practice).

A pilot study involving 30 nursing assistants demonstrated good preliminary internal consistency (Cronbach’s *α* = 0.805). In the main study, the overall scale demonstrated excellent internal consistency (Cronbach’s *α* = 0.895). The internal consistency for the three subscales was also assessed, yielding the following Cronbach’s α coefficients: Knowledge = 0.676 (indicating acceptable but lower reliability compared to the other subscales), Attitude = 0.914, and Practice = 0.862, indicating acceptable to excellent reliability. Furthermore, the construct validity of the scale was examined using exploratory factor analysis (EFA). The Kaiser-Meyer-Olkin (KMO) measure of sampling adequacy was 0.895, and Bartlett’s test of Sphericity was significant (2 = 10105.742, *p* < 0.001), confirming the data’s suitability for EFA. Principal axis factoring with Promax rotation was conducted, which extracted three factors with eigenvalues greater than 1. These three factors collectively explained 62.666% of the total variance, providing empirical support for the proposed three-dimensional structure of the KAP assessment tool.

### 2.4 Data collection

After obtaining administrative approval from participating institutions, the survey was conducted with 30 nursing assistants to refine the questionnaire’s clarity and feasibility. The finalized questionnaire was administered online using the Wenjuanxing (Questionnaire Star) platform. To ensure a high response rate and data quality, a multi-step procedure was implemented: (1) the survey link was distributed by the heads of nursing departments or third-party agency managers to all eligible nursing assistants within their units, accompanied by a personal appeal emphasizing the study’s importance for occupational safety; (2) two reminder messages were sent via the same internal channels at one-week intervals to encourage participation; and (3) participation was explicitly presented as voluntary and anonymous to minimize social pressure. Submitted responses were screened for quality. Invalid questionnaires (e.g., completion time <1 min, straight-lining, or with key demographic data missing) were excluded, resulting in 503 valid responses from 505 initial submissions.

### 2.5 Statistical analysis

We performed all statistical analyses using SPSS (version 26.0; IBM Corp., Armonk, NY, United States). Descriptive statistics are presented as frequencies and percentages for categorical variables and as means ± standard deviations for continuous variables. For each item and dimension, we calculated a score rate (mean score divided by maximum possible score) and expressed it as a percentage. We determined the optimal number of clusters (k) by identifying the inflection point in the plot of within-cluster variance against k using the elbow method. To provide statistical validation for the cluster solution, we also calculated the average silhouette width and the Calinski-Harabasz index for cluster solutions ranging from k = 2 to k = 8. Prior to clustering, the raw scores of these three dimensions were standardized into Z-scores to account for their differing measurement scales (Knowledge: 0–15; Attitude and Practice: 0–75) and to ensure each dimension contributed equally to the cluster solution. To identify factors associated with cluster membership, we performed univariate analyses using the chi-square test for categorical variables and the Kruskal-Wallis H test for continuous variables, as appropriate. Variables significantly associated with cluster membership in the univariate analyses (*p* < 0.05) were entered as candidates into a multinomial logistic regression model; the Synergistic KAP Group was designated as the reference category for the multinomial logistic regression analysis. Model fit goodness will be evaluated using the likelihood ratio test and pseudo R^2^ indices. Multicollinearity was assessed using Variance Inflation Factors (VIF). This model identified independent factors associated with membership in the different KAP clusters. A two-sided *p*-value of < 0.05 was considered statistically significant for all analyses. To verify the robustness of the four-cluster solution, we further conducted three complementary analyses, including random seed variation, random data subset sampling, and alternative distance metric comparison, with the Adjusted Rand Index (ARI) used to quantify the consistency of clustering results.

## 3 Results

### 3.1 Demographic characteristics

Of the 505 questionnaires distributed, 503 were included in the final analysis after excluding two with incomplete key data, yielding an effective response rate of 99.6%. The mean age of participants was 49.0 ± 7.11 years. The sample comprised 35 males (7.0%) and 468 females (93.0%). Most participants (60.0%) had attained an education level of junior high school or lower (Table 1).

**Table 1:** Demographic characteristics.

Variables	*N*(%)
Gender	
Male	35(7.0)
Female	468(93.0)
Years of experience (year)	
<3	198(39.4)
3 ~ 5	90 (17.9)
>5	215 (42.7)
Education	
Primary school or below	61 (12.1)
Junior middle school	335 (66.6)
Senior high school	87 (17.3)
College or above	20 (4.0)
Type of healthcare institution	
Comprehensive tertiary hospital	468 (93.0)
Comprehensive secondary hospital	35 (7.0)
Department	
Internal medicine	151 (30.0)
Surgery	164 (32.6)
Other	188 (37.4)
Average monthly income (CNY)	
<6,000	273 (54.3)
6,000 ~ 8,000	206 (41.0)
>8,000	24 (4.7)
Received systematic occupational protection training	
Yes	493 (98.0)
No	10 (2.0)
Holds nursing assistants certification	
Yes	385 (76.5)
No	118 (23.5)

### 3.2 Occupational protection knowledge

The mean knowledge score was 13.78 ± 1.71, corresponding to a score rate of 91.9%. Across the knowledge sub-dimensions, only “Personal Basic Protection” had a score rate below 90%. At the item level, correct response rates ranged from 78.3 to 99.6%. Four items had a correct response rate below 90%: item 3 (78.3%), item 4 (88.1%), item 5 (87.3%), and item 7 (88.1%) (Tables 2, 3).

**Table 2:** Knowledge of nursing assistants.

Dimension	Total score	Score (mean ± SD)	Score rate (%)
Personal basic protection	3	2.65 ± 0.58	88.3
Biological protection	3	2.71 ± 0.56	90.3
Physical protection	3	2.76 ± 0.55	92.0
Chemical protection	3	2.83 ± 0.44	94.3
Psychosocial protection	2	1.83 ± 0.45	91.5
Learning and supervisory behavior	1	0.99 ± 0.06	99.0

**Table 3:** Bottom 4 items by score on occupational protection knowledge.

Item	Correct [*N*(%)]	Incorrect [*N*(%)]
3. Steps of the seven-step handwashing method: ① Palm; ② Back of fingers and finger gaps; ③ Palm-side finger gaps; ④ Back of fingers; ⑤ Thumbs; ⑥ Finger tips; ⑦ Wrists and forearms.	394 (78.3)	109 (21.7)
4. All patients and hospital bed units should be regarded as potentially infectious and protected accordingly.	443 (88.1)	60 (11.9)
5. Wearing gloves during operations that may involve contact with patient blood or body fluids can reduce occupational exposure.	439 (87.3)	64 (12.7)
7. Wearing compression stockings during prolonged standing can effectively relieve or reduce the pressure on lower extremity veins and venous valves, and prevent varicose veins.	443 (88.1)	60 (11.9)

### 3.3 Occupational protection attitude

The mean attitude score was 65.90 ± 5.54 (score rate: 87.9%), reflecting a generally positive attitude toward occupational protection. The lowest-scoring items were 7, 8, and 14 (Tables 4, 5).

**Table 4:** Attitude of nursing assistants.

Dimension	Total score	Score (mean ± SD)	Score rate (%)
Personal basic protection	15	14.42 ± 1.06	96.1
Biological protection	15	14.18 ± 1.33	94.5
Physical protection	15	13.67 ± 1.84	91.1
Chemical protection	15	14.37 ± 1.20	95.8
Psychosocial protection	10	9.30 ± 1.17	93.0
Learning and supervisory behavior	5	4.79 ± 0.47	95.8

**Table 5:** Bottom 3 items by score on occupational protection attitude.

Item	Total score	Score (mean ± SD)	Score rate (%)
7. Do you think it is important to wear compression stockings during prolonged standing to reduce occupational hazards?	5	4.39 ± 0.91	87.8
8. Do you think it is important to keep elbows as close to the body as possible when lifting heavy objects at work to reduce occupational hazards?	5	4.57 ± 0.71	91.4
14. Do you think it is important to communicate with others, confide in inner troubles, and seek advice when encountering setbacks, frustrations, or difficulties to maintain physical and mental health?	5	4.59 ± 0.76	91.8

### 3.4 Occupational protection practices

The mean practice score was 63.23 ± 8.80, yielding a score rate of 84.3%. Notably, the “Physical Protection” (70.9%) and “Psychosocial Protection” (77.1%) sub-dimensions had the lowest score rates. The lowest-scoring practice items were 7, 8, 9, 13, and 14 (Tables 6, 7).

**Table 6:** Practices of nursing assistant.

Dimension	Total score	Score (mean ± SD)	Score rate (%)
Personal basic protection	15	13.48 ± 1.89	89.9
Biological protection	15	13.26 ± 1.87	88.4
Physical protection	15	10.63 ± 3.08	70.9
Chemical protection	15	13.73 ± 1.96	91.5
Psychosocial protection	10	7.71 ± 2.28	77.1
Learning and supervisory behavior	5	4.42 ± 0.84	88.4

**Table 7:** Bottom 5 items by score on occupational protection practices.

Item	Total score	Score (mean ± SD)	Score rate (%)
7. Do you always wear compression stockings during prolonged standing?	5	2.85 ± 1.50	57.0
8. Do you keep elbows as close to the body as possible when lifting heavy objects at work?	5	3.87 ± 1.12	77.4
9. At work, do you pay attention to avoiding prolonged same postures, move the neck and legs frequently, and reduce prolonged waist stretching and bending?	5	3.91 ± 1.06	78.2
13. When conflicts arise with others (patients, family members, medical staff, etc.) and the situation cannot be eased quickly, do you leave the scene and seek help from security personnel or superiors?	5	3.92 ± 1.30	78.4
14. When encountering setbacks or difficulties, do you communicate with others, confide in inner troubles, and seek advice?	5	3.79 ± 1.26	75.8

### 3.5 Comparison of occupational protection KAP cluster scores among nursing assistants

**Figure 1:**
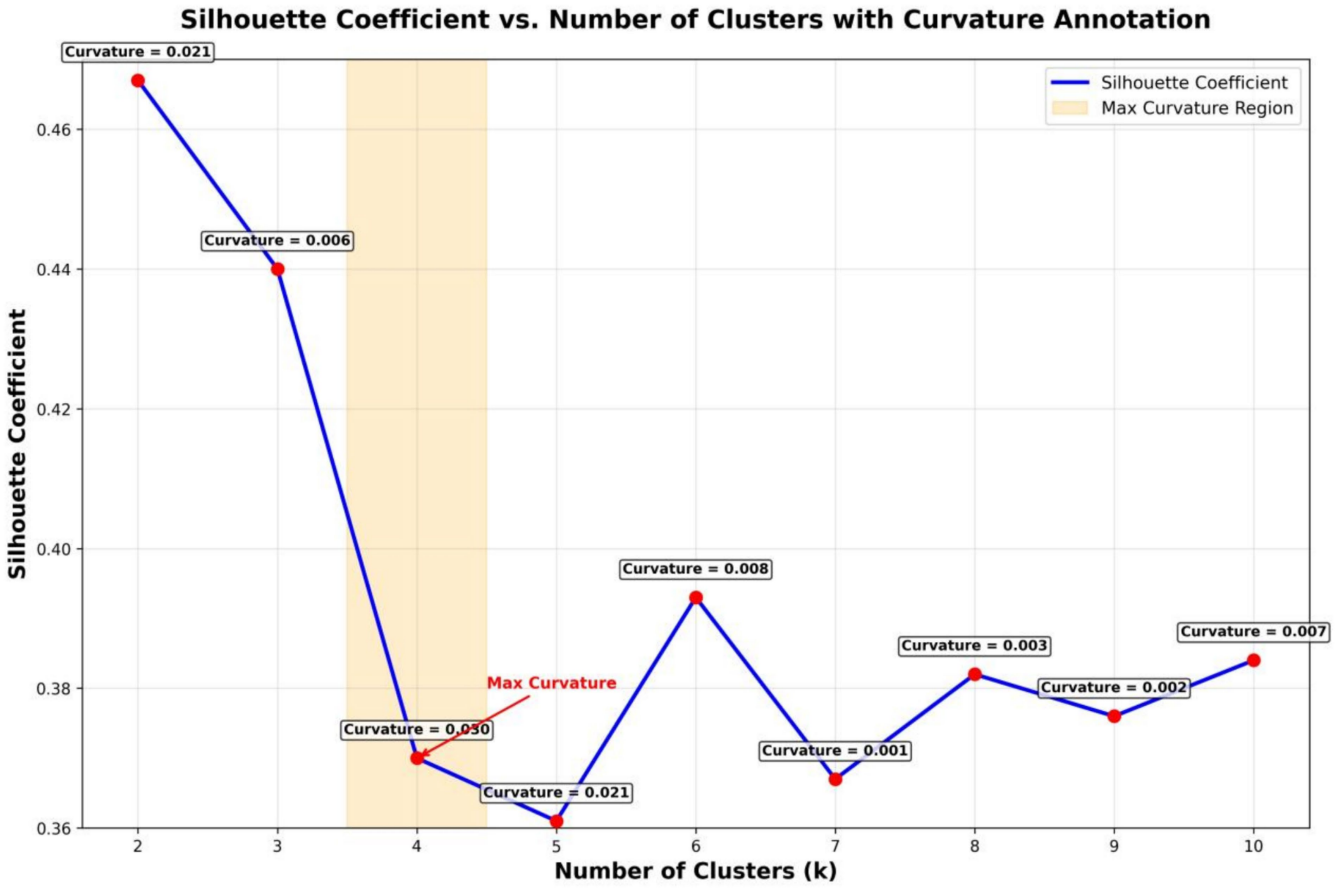
Determination of the optimal number of clusters (elbow method).

To identify distinct profiles among participants, a K-means cluster analysis was performed on the standardized total scores from the KAP scale. The optimal number of clusters was determined using the elbow method (see Figure 1), which identifies the point where the reduction in within-cluster variance begins to diminish markedly. A four-cluster solution (k = 4) was selected based on the elbow method output, cluster interpretability, practical relevance, and consultations with subject-matter experts.

This analysis classified the 503 nursing assistants into four distinct clusters based on their standardized KAP dimension scores. The clusters were labeled: (1) Synergistic Group (47.3%), (2) Weak KAP Group (10.1%), (3) High-Knowledge–Low-Attitude–Weak-Practice Group (18.7%), and (4) High-Knowledge–High-Attitude–Low-Practice Group (23.9%) (Table 8).

**Table 8:** Scores on KAP dimensions across different clusters of nursing assistants.

Cluster	Knowledge (mean ± SD)	Attitude (mean ± SD)	Practice (mean ± SD)
Synergistic KAP group	14.37 ± 1.02	69.17 ± 1.70	70.12 ± 3.42
Weak KAP group	10.08 ± 1.75	59.53 ± 6.34	55.75 ± 8.36
High-knowledge-low attitude-weak practice group	14.12 ± 0.96	57.94 ± 3.03	58.16 ± 7.77
High-knowledge-high attitude-low practice group	13.90 ± 1.29	68.35 ± 2.13	56.52 ± 6.29

#### 3.5.1 Comparison of demographic characteristics across clusters

Demographic characteristics were compared across the four identified clusters. Statistically significant differences (*p* < 0.05) were observed in the type of employing institution, possession of a professional qualification certificate, and age.

Regarding the type of institution, the proportion of nursing assistants working in tertiary grade A hospitals was highest in the Synergistic KAP Group (96.6%) and lowest in the High Knowledge–High Attitude–Low Practice Group (82.4%). Concerning professional qualification, the certification rate was highest in the High Knowledge–High Attitude–Low Practice Group (86.3%) and lowest in the High Knowledge–Low Attitude–Weak Practice Group (66.0%) (Table 9).

**Table 9:** Comparison of demographic characteristics across clusters.

Variables	Synergistic KAP group [*N*(%)]	Weak KAP group [*N*(%)]	High-knowledge-low attitude-weak practice group [*N*(%)]	High-knowledge-high attitude-low practice group [*N*(%)]	*p*
Gender					
Male	16 (6.7)	4 (7.8)	4 (4.3)	11 (9.2)	0.563
Female	222 (93.3)	47 (92.2)	90 (95.7)	109 (90.8)	
Years of experience (year)					
<3	84 (35.3)	27 (52.9)	39 (41.5)	48 (40.0)	0.198
3 ~ 5	44 (18.5)	11 (21.6)	16 (17.0)	19 (15.8)	
>5	110 (46.2)	13 (25.5)	39 (41.5)	53 (44.2)	
Education					
Primary school or below	33 (13.9)	4 (7.9)	11 (11.7)	13 (10.3)	0.779
Junior middle school	151 (63.4)	35 (68.6)	68 (72.3)	81 (67.5)	
Senior high school	46 (19.3)	10 (19.6)	10 (10.6)	21 (17.5)	
College or above	8 (3.4)	2 (3.9)	5 (5.4)	5 (4.2)	
Type of healthcare institution					
Comprehensive tertiary hospital	230 (96.6)	42 (82.4)	90 (95.7)	106 (88.3)	<0.01
Comprehensive secondary hospital	8 (3.4)	9 (17.6)	4 (4.3)	14 (11.7)	
Department					
Internal medicine	74 (31.1)	14 (27.5)	32 (34.0)	32 (26.6)	0.838
Surgery	75 (31.5)	15 (29.4%)	30 (31.9)	44 (36.7)	
Other	89 (37.4)	22 (43.1%)	32 (34.0)	44 (36.7)	
Average monthly income (CNY)					
<6,000	124 (52.1)	31 (60.8)	56 (59.6)	62 (51.7)	0.473
6,000 ~ 8,000	101 (42.4)	16 (31.4)	35 (37.2)	54 (45.0)	
>8,000	13 (5.5)	4 (7.8)	3 (3.2)	4 (3.3)	
Received systematic occupational protection training					
Yes	234 (98.3)	50 (98.0)	92 (97.9)	117 (97.5)	0.963
No	4 (1.7)	1 (2.0)	2 (2.1)	3 (2.5)	
Holds nursing assistant certification					
Yes	187 (78.6)	44 (86.3)	62 (66.0)	92 (76.7)	0.028
No	51 (21.4)	7 (13.7)	32 (34.0)	28 (23.3)	
Age (year)	47.94 ± 8.03	49.67 ± 6.51	50.90 ± 5.48	48.92 ± 7.25	<0.01

#### 3.5.2 Multivariate logistic regression analysis of factors associated with KAP clusters

Categorical variables were coded as dummy variables for the analysis: type of institution was coded with “secondary grade A hospitals” as the reference category (tertiary grade A hospitals = 1); possession of a professional qualification certificate was coded with “no” as the reference (yes = 1). Age was entered as a continuous variable. The Synergistic KAP Group was specified as the reference category. The results indicated that the type of institution and possession of a professional qualification certificate were significant factors influencing the different KAP clusters among nursing assistants (Table 10). The likelihood ratio test shows that the final model significantly outperforms the null model (2(9) = 43.312, *p* < 0.001).

**Table 10:** Multivariate analysis of factors associated with different KAP clusters.

Item		Weak KAP group		High knowledge-low attitude-weak practice group		High knowledge-high attitude-low practice group		Collinearity statistics	
		OR	95% CI	OR	95% CI	OR	95% CI	Toler-ance	VIF
Type of healthcare institution	Comprehensive tertiary hospital	0.136^*^	0.048 ~ 0.382	0.918	0.261 ~ 3.224	0.260^*^	0.105 ~ 0.647	0.967	1.034
Holds nursing assistants certification	Yes	1.969	0.799 ~ 4.852	0.422^*^	0.242 ~ 0.736	0.919	0.530 ~ 1.596	0.984	1.016
Age (year)		1.027	0.981 ~ 1.075	1.073	0.033 ~ 1.115	1.025	0.993 ~ 1.058	0.953	1.049

^*^p<0.05. Cox and Snell pseudo R^2^ = 0.083, Nagelkerke pseudo R^2^ = 0.090, and McFadden pseudo R^2^ = 0.035 for the ordinal logistic regression model.

#### 3.5.3 Cluster validity and robustness analysis

The quality of the four-cluster solution was assessed using three internal validity metrics: the Silhouette Coefficient (0.3699), the Davies-Bouldin Index (0.82), and the Calinski-Harabasz Index (128.6). A Silhouette Coefficient of 0.3699 indicates moderate to weak cluster separation. While the clusters demonstrate some structural validity, this suggests a degree of inter-cluster overlap, highlighting the complexity of the KAP profiles among nursing assistants. We further verified the robustness of the four-cluster solution using three classical approaches: random seed variation (10 seeds), 80% random data subset sampling (5 replicates), and alternative distance metric comparison (Manhattan/cosine vs. Euclidean). The Adjusted Rand Index (ARI) was all > 0.82 across all analyses, and the variation in cluster proportion was less than 3%, indicating high stability and generalizability of the four-cluster solution. These results confirmed that the identified KAP profiles reflected the intrinsic heterogeneity of the study population rather than random clustering outcomes.

## 4 Discussion

### 4.1 A KAP conversion funnel exists, with practice as the weak link

The surveyed nursing assistants demonstrated a moderately high overall occupational protection KAP score (142.90 ± 12.94). Scores for the individual dimensions were as follows: knowledge (13.78 ± 1.71; 91.9%), attitude (65.90 ± 5.54; 87.9%), and practice (63.23 ± 8.80; 84.3%). This pattern of descending mean scores across dimensions suggests a potential misalignment or gap between high knowledge, positive attitudes, and protective practices. Analysis of the lowest-scoring items (Tables 3, 5, 7) highlights specific domains where performance is notably weaker across dimensions. While knowledge deficits were primarily procedural (Table 3), the lowest scores were concentrated in physical and psychosocial protection items (Table 7), which also showed relatively lower attitude scores (Table 5). This finding corroborates prior research (18) and underscores the multifactorial complexity of behavioral adoption, which extends beyond knowledge and attitudes to include factors such as educational background and systemic supports (19). A key explanatory factor may be their frequent employment through third-party agencies, which can diminish professional identity and organizational belonging (20, 21). Consequently, healthcare institutions may under-invest in protective resources, systematic training, and performance feedback mechanisms for this perceived external workforce (22). This support deficit ultimately undermines the conditions necessary to translate knowledge into sustained practice.

Notably, the consistently low scores in physical and psychosocial protection suggest that current protocols may prioritize traditional biological and chemical risks while overlooking other critical threats (23). Therefore, a shift from a “single-dimensional technical” approach to a “systematic prevention and control management” model is warranted. This entails establishing supportive organizational environments and developing integrated training systems (24, 25) to enable behavioral transformation.

### 4.2 Group-specific patterns exist in occupational protection KAP among nursing assistants

K-means cluster analysis identified four distinct profiles: the Synergistic KAP Group (47.3%), the Weak KAP Group (10.1%), the High-Knowledge–Low-Attitude–Weak-Practice Group (18.7%), and the High-Knowledge–High-Attitude–Low-Practice Group (23.9%). This heterogeneity necessitates tailored interventions. The Synergistic KAP Group demonstrated the highest scores across all three dimensions—knowledge, attitude, and practice. For this group, management efforts should focus on maintenance and enhancement. Establishing “model protection positions” or selecting peer educators (26) could leverage their exemplary role to positively influence. The Weak KAP Group scored the lowest in both knowledge and practice. This group is at high risk for occupational exposure. Management should prioritize this group by providing foundational training that emphasizes basic knowledge and fosters a core belief in the importance of protection. The High-Knowledge-Low-Attitude-Weak-Practice group scored the lowest in occupational protection attitude among the four clusters. Interventions for this group should focus on transforming attitudes by employing real-life case warnings of occupational exposure (27) and science-based education on occupational health hazards. These approaches aim to dispel evidence-based complacency, strengthen the understanding of the direct link between protection practices and personal health, promote a shift toward more positive protective attitudes, and thereby drive behavioral improvements. The High-Knowledge-High-Attitude-Low-Practice Group exhibited the lowest occupational protection practice score among the four clusters. The lag in their protective behaviors is not due to unwillingness but may be directly related to systemic issues such as work overload, fragmented workflows, and insufficient resource support (28). This suggests that management should focus on addressing the obstacles in translating knowledge and attitude into behavior, optimizing the work environment, and allocating human resources rationally, standardizing operating procedures (29).

### 4.3 Multiple factors influence the KAP cluster types of nursing assistants

The multivariate analysis revealed that institution type and possession of a professional qualification certificate significantly influenced the formation of distinct KAP clusters among nursing assistants, with each factor impacting the KAP conversion process through different pathways.

The proportion of nursing assistants classified into the Synergistic KAP Group was significantly higher in tertiary hospitals (96.6%) than in secondary hospitals (3.4%). Furthermore, the analysis indicated that nursing assistants in tertiary hospitals had a lower risk of belonging to the Weak KAP Group. This disparity could potentially be associated with the frequently more robust occupational protection management systems, standardized training protocols, and adequate resource allocation characteristic of many tertiary hospitals (30). Moreover, stringent daily supervision and evaluation in these institutions could foster a supportive systemic environment and practical atmosphere that facilitates the translation of protective knowledge into actual behavior. Conversely, secondary hospitals might have relatively less developed systemic support for nursing assistants’ protective practices, which could be associated with relatively limited resource allocation, insufficient frequency and depth of protection training, and less robust monitoring mechanisms (31), which could present additional challenges to the synergistic development of their KAP. Given that our sample predominantly comprised tertiary hospital staff, these institutional comparisons should be interpreted cautiously.

Possession of a professional qualification certificate was associated with a markedly lower risk of belonging to the High-Knowledge–Low-Attitude–Weak-Practice Group. This suggests that systematic certification training effectively consolidates knowledge and reinforces the health relevance of protection (3). Promoting certification and practical training within it is therefore a key strategy.

This study did not identify a significant influence of age. This likely indicates that its initial association with cluster membership was confounded by the more influential structural factors of institution type and professional certification. The data suggest that age itself is not a primary driver of KAP patterns; rather, the systemic support and formal training embedded in these structural factors are the key determinants. The underlying mechanisms warrant further exploration in future research.

Furthermore, the modest model fit, indicated by the low pseudo R^2^ values (e.g., McFadden = 0.035), suggests limited explanatory power. Although the institutional and certification effects are statistically significant, they should not be overinterpreted. These factors are part of a more complex explanatory web, and future research should investigate additional mediators such as organizational culture and individual characteristics.

### 4.4 Strengths and limitations of the work

The findings of this study indicate that nursing assistants demonstrated a satisfactory overall level of occupational protection, characterized by relatively good knowledge but lagging attitudes and practices, demonstrating a knowledge-attitude-practice conversion funnel. Cluster analysis identified four distinct KAP profiles: the Synergistic KAP Group, the Weak KAP Group, the High-Knowledge–Low-Attitude–Weak-Practice Group, and the High-Knowledge–High-Attitude–Low-Practice Group. Significant heterogeneity was observed among these profiles, with membership showing statistically significant associations (*p* < 0.05) with the type of healthcare institution and possession of a professional qualification certificate.

This study has several limitations. First, the sample’s recruitment through third-party agencies may introduce selection biases (e.g., age), potentially limiting its representativeness of the broader nursing assistant population. Second, participants were predominantly from tertiary hospitals (93%). This distribution primarily reflects the operational scope of the collaborating third-party agencies, whose services are concentrated in tertiary hospitals, and the clinical reality that these hospitals, as regional referral centers, manage a higher volume of complex cases. While this sample accurately represents nursing assistants in core, high-acuity settings and allows for an in-depth analysis of KAP within such environments, it limits the generalizability of findings—particularly those related to institutional-level effects—to secondary or community hospitals. Future studies should employ stratified sampling to ensure balanced representation across healthcare institution tiers. Third, the cross-sectional design precludes causal inferences regarding the relationships among knowledge, attitude, and practice; longitudinal or intervention studies are required to establish directional pathways. Fourth, the Cronbach’s alpha for the Knowledge subscale was 0.676, which lies at the lower boundary of acceptable reliability. This may be attributed to the binary scoring format or the specific construct of the knowledge items; future studies could refine this subscale to enhance its internal consistency. Finally, the low variability in the “received systematic training” variable (98% “yes”) constrains the analytical power to detect its potential associations with KAP outcomes, suggesting that future research should employ more nuanced measures of training exposure.

### 4.5 Recommendations for further research

Future research should employ multi-center collaboration and stratified sampling to enhance sample representativeness and further explore the factors influencing the occupational protection KAP of nursing assistants.

### 4.6 Implications for policy and practice

In conclusion, our findings suggest the potential value of a paradigm shift in managing nursing assistants’ occupational protection. Moving beyond one-size-fits-all training, institutions could consider adopting a precision public health approach. This precision approach entails two core strategies: first, leveraging KAP profiling to inform resource allocation, with a particular focus on enhancing systemic supports in secondary hospitals; second, exploring the integration of KAP evaluations and competency-based training into the contractual frameworks of third-party agencies and institutional quality assurance systems. To address the disparities observed, future policy development might prioritize the equitable allocation of dedicated training resources. Furthermore, strengthening and promoting the professional certification system could be a promising strategy to build a foundational level of competence. Ultimately, protecting this critical workforce is likely to require shared responsibility among stakeholders to create environments that support safe practices.

## 5 Conclusion

Nursing assistants demonstrated good knowledge of occupational protection, but there was a notable gap in practical implementation. The four KAP types identified through cluster analysis showed high heterogeneity. Targeted interventions should be developed based on these distinct profiles, shifting the focus from individual training to the establishment of systematic and supportive organizational environments and management systems.

## Data Availability

The original contributions presented in the study are included in the article/supplementary material, further inquiries can be directed to the corresponding authors.
